# The effect of the various doses of atorvastatin on renal tubular cells; an experimental study

**DOI:** 10.15171/jnp.2016.20

**Published:** 2016-02-14

**Authors:** Hamid Nasri, Zahra Hasanpour, Mehdi Nematbakhsh, Ali Ahmadi, Mahmoud Rafieian-Kopaei

**Affiliations:** ^1^Department of Internal Medicine, Isfahan University of Medical Sciences, Isfahan, Iran; ^2^Water and Electrolytes Research Center, Isfahan University of Medical Sciences, Isfahan, Iran; ^3^Department of Epidemiology and Biostatistics, Shahrekord University of Medical Sciences, Shahrekord, Iran; ^4^Medical Plants Research Center, Shahrekord University of Medical Sciences, Sharekord, Iran

**Keywords:** Atorvastatin, HMG-COA reductase inhibitors, Renal toxicity, Rhabdomyolysis

## Abstract

**Background:**

Recent retrospective observational studies suggest that high-potency statin therapy might increase the risk of acute kidney injury, however data on this subject is scares.

**Objectives:**

This study, was designed to investigate the renal tubular cell effect of different doses of atorvastatin to detect the possible aggravation of renal function or morphology of the kidney.

**Materials and Methods:**

In this experimental study 24 male Wistar rats were designated into 4 equal groups and treated as follows. Control group received phosphate buffer as the vehicle of atorvastatin for 7 days. Groups 1, II and III received atorvastatin at doses of 10, 50 and 150 mg/kg daily for 7 days, then on the day 8, all rats were anesthetized using ketamine and the blood samples were collected for evaluation of creatinine (Cr) and blood urea nitrogen (BUN) levels and then all rats were sacrificed, then the animals’ kidneys were dissected out and histopathological studies were performed.

**Results:**

Mean (±SD) of scores of injury to renal tubular cells in control group was 4.2 ± 2.2 and in groups I, II and III were 6.44 ± 4.9, 15.4 ± 8.5 and 25.8 ± 12.7 respectively. Group III which received 150 mg/kg/day of atorvastatin had significant renal damage in comparison to control group (*P* < 0.001). There was no significant difference of renal injury score between control group with groups of I and II.

**Conclusions:**

In the present study we found, atorvastatin with a dose of 150 mg/kg/day for 7 days was nephrotoxic for rats, while lower doses at 10 mg/kg/day or 50 mg/kg/day for 7 days were not accompanied by renal injury. These findings imply further attention to the administration of higher doses of atorvastatin in clinical conditions.

**Implication for health policy/practice/research/medical education:**

In an experimental study, we found, atorvastatin with a dose of 150 mg/kg/day for 7 days was nephrotoxic for rats, while lower doses at 10 mg/kg/day or 50 mg/kg/day for 7 days were not accompanied by renal injury. These findings imply further attention to the administration of higher doses of atorvastatin in clinical conditions.

## 1. Background

Since the discovery of 3-hydroxy-3-methylglutaryl coenzyme A (HMG-CoA) reductase inhibitors (statins) in 1976, they have been described as the principal and the most effective agents for reducing serum cholesterol values (1,2). Statins have proven their beneficial effects in reducing total and LDL-cholesterol as well as triglycerides, and in increasing HDL-cholesterol (2).Statins are amongst the most widely recommended drugs, with established benefits in patients at risk of heart disease. Statins have had a dramatic impact on the primary and secondary prevention of cardiovascular disease. However, the efficiency and relative safety of HMG-CoA (3-hydroxy-3-methylglutaryl coenzyme A) reductase inhibitors, has permitted many to argue for universal statin administration in everyone older than 50 years (3,4).While, administration of high-potency statins has been detected to be more efficient in improving cardiovascular outcomes than use of lower-potency statins, but, recent concerns about the risks of rhabdomyolysis and acute kidney injury, diabetes mellitus, and memory impairment have prompted some investigators to detect more precisely the side effects of statins (5). Indeed, a few retrospective observational studies suggest that high-potency statin therapy might increase the risk of acute kidney injury, however data on this subject is scares (6).

## 2. Objectives

This study therefore, was designed to investigate the renal tubular cell effect of different doses of atorvastatin to detect the possible aggravation of renal function or morphology of the kidney.

## 3. Materials and Methods

### 3.1. Animals

In this experimental study 30 male Wistar rats weighing 200-250 g were selected and similarly handled in the animal house of Isfahan University of Medical Sciences, Isfahan, Iran. The animals were kept at a controlled environment with 50%-60% humidity and temperature of 25 ± 3°C. Additionally, the rats were housed with a 12 hours dark-light cycle (lights on at 7:00 am) and permitted free access to pelleted diet and tap water. They were also housed in animal lab at least one week prior to the experiment. During the investigation, the animal’s general health state and activity were monitored closely. The project was confirmed by the Ethical Committee of Isfahan University of Medical Sciences, Isfahan, Iran and all animal experimentations were performed in accordance with the National Institute of Health guidelines for the careful use of laboratory animals.

### 3.2. Drugs and chemicals

Atorvastatin was prepared from Kharazmi Pharmaceutical Company (Tehran, Iran), and administered intraperitoneally (i.p.).

### 3.3. Experimental design

In this experimental study 24 male Wistar rats were designated into 4 equal groups and treated as follows. Control group received phosphate buffer as the vehicle of atorvastatin for 7 days. Groups 1, II and III received atorvastatin at doses of 10, 50 and 150 mg/kg daily for 7 days, then on the day 8, all rats were anesthetized using ketamine and the blood samples were collected for evaluation of creatinine (Cr) and blood urea nitrogen (BUN) levels and then all rats were sacrificed.

### 3.4. Histopathological examination

At the end of the experiment the animals’ kidneys were dissected out and fixed in buffered formalin for 12 hours and processed for histopathological examinations. Three μm-thick paraffin sections were stained with Hematoxylin and Eosin (H&E) for light microscopic examination using conventional protocol.

Histopathological studies were performed under a light microscope. Slides were coded and examined by a nephropathologist who was blinded to the treatment groups. All specimens were examined for morphologic parameters including epithelial cell degeneration, vacuolization, tubular dilatation, tubular cell flattening and presence of hyaline cast and debris materials in tubular lumen.

### 3.5. Statistical analysis

All Numerical variables with a normal distribution were expressed as mean ± SD and categorical variables are presented as percentage. According to normal data distribution, one-way analysis of variance (ANOVA) and post hoc tests (Bonferroni test) were used for the comparison of mean values between groups. A new variable of score was generated by means of histopathology evaluations of degeneration, vacuolization, tubular dilatation, tubular cell flattening and presence of hyaline cast and debris of injury to renal tubular cells. *P* values of less than 0.01 were assumed to be significant (*P*<0.01). Data analysis was done using Stata software (Release 12, Stata Corp., College Station, TX).

## 4. Results

Mean (±SD) of scores of injury to renal tubular cells in control group was 4.2±2.2 and in groups I, II and III were 6.44±4.9, 15.4±8.5 and 25.8±12.7 respectively. Mean (±SD) of scores of injury to renal tubular cells was illustrated in Table 1. Group III which receive 150 mg/kg/day of atorvastatin had significant renal damage in comparison to control (buffer) group (*P*<0.001). There was no significant difference of renal injury score between control group with groups of I and II. Comparison between groups was summarized in Tables 2 and 3.

**Table 1:** Mean ± SD of dilatation , degeneration, vacuolization and debris of renal tubular cells

**Groups**	**Vacuolization**	**Degeneration**	**Debris**	**Dilatation**	**Score**
Control: 0	10±5	10.6±5.5	2.31±1.5	1.3±0.7	4.2±2.2
I: at 10	15±12	15±11	3.5 ±1.6	2.3±2.2	6.44±4.9
II: at 50	34±15	24.6±15.7	18.3±7.5	6.3±9	15.4±8.5
III: at 150	55±25	50±22.8	22.5±13	9.5±6	25.8±12.7
Total	28.5±23.6	25.08±21	11.6±11	4.8±6.4	13±11.5
F statistic	9.55	8.02	10.56	2.47	8.78
*P* value	0.0001	0.002	0.001	0.09	0.001

**Table 2:** Comparison of dilatation, degeneration, vacuolization and debris of renal tubular cells between groups

**Between groups comparison**	**Vacuolization**	**Degeneration**	**Dilatation**	**Score**
0 versus I	5	4.3	1.01	2.1
0 versus II	24.1	14	5.01	11.1
0 versus III	45*	39.3*	8.1	21.6*
I versus II	19.1	9.6	4	8.9
I versus III	40*	35*	7.1	19.4*
II versus III	20.8	25.3	3.1	10.4

*P values of less than 0.01 were assumed to be significant (*P*<0.01)

**Table 3:** Mean ± SD of Cr

**Groups**	**Cr**
0: Buffer	0.53±0.03
I: at 10	0.5±0.0
II: at 50	0.53±0.05
III: at 150	0.55±0.05
Total	0.52±0.04
F statistic^a^	1.49
*P* value	0.246

^a^One-way ANOVA for comparison groups.

## 5. Discussion

In the present study we found, atorvastatin with a doses of 150 mg/kg/day for 7 days was nephrotoxic for rats. While lower doses at 10 mg/kg/day or 50 mg/kg/day for 7 days was not accompanied by renal injury.

Inflammation is highly prevalent in patients with chronic renal failure and is consistently associated with cardiovascular mortality and morbidity (7).Dyslipidemia are encountered as the major risk factor for atherosclerosis and its associated conditions like coronary artery disease, peripheral vascular disease and ischemic cerebrovascular disease (7).One of the drugs that are available to treat dyslipidemias consist the statins (3-hydroxy-3-methylglutaryl coenzyme A reductase inhibitors).While a bulk of clinical evidences has demonstrated that statin therapy significantly reduces the risk of new or recurrent cardiovascular events and stroke, and ameliorates survival of individuals with previous cardiovascular disease (1-3), however, clinical trials of transplant survival reveal the therapeutic potential of statins as immunosuppressive agents. Statins protect against rejection of cardiac transplantations and may be beneficial in pulmonary and kidney transplantation. Interestingly, some investigations support the protective role of statins in heart graft survival. The beneficial properties of statins implies modification of endothelial function, plaque stability, and, specially, inflammatory pathways. These properties are addressed to as ‘pleiotropic effects’ of statins. Existence of these pleiotropic actions reveals that statins might be beneficial in the treatment of patients with chronic renal failure. In addition, statins are able to act in the kidney as a potent free radical scavenger and inhibit mitogen activated protein kinase and nuclear factor kappa B. Statins also are capable to inhibit signaling pathways activation by reactive oxygen species and therefor prevents tubular cell apoptosis induced by various nephrotoxins (4,5).Additionally statins affect, various signaling pathways involving renal inflammatory, proliferative, and cell-death responses. Hence, statins exert anti-inflammatory actions in renal tissue (6). Moreover, renal antioxidant efficacies with subsequent endothelial function regulation of renal vasculature, after statin administration, may also explain the pleiotropic protection against renal injury. Conversely various recent investigations had indicated that administration of statins in high doses may itself directed to renal tubular cell injury (6). In a study on 56 male Sprague Dawley rats, Reddy et al found that, high dose of atorvastatin + garlic extract has negative safety profile when compared with groups having low dose of statin and high dose of garlic juice (8). They interpreted that, high dose of atorvastatin induces damage to the kidney whether used either alone or in combination with high concentration of garlic, whereas, low dose of atorvastatin in combination with high concentration of garlic juice has minimal nephrotoxic potential (8). Studies have shown, high-potency statins has been to be more effective in improving cardiovascular outcomes than use of lower-potency statins. However, recent findings suggest that high-potency statin therapy may increase the risk of acute kidney injury (9).To compare the impact of treatment with high-potency versus low-potency statins on risk of acute kidney injury, Dormuth et al, analyzed the administrative healthcare records of more than two million people aged ≥40 years who received new prescriptions for statins during the investigation period (10). They detected that during the first 120 days of therapy, patients without chronic renal failure who received high-potency statins had a 34% higher risk of acute kidney injury hospitalizations than those who received low-potency statins. This risk remained increased for at least the first two years of statin treatment. In patients with chronic renal failure, the use of high-potency versus low-potency statins, did not significantly increase the risk of hospitalization for acute kidney injury (10). They concluded that high-potency statin therapy may increase the risk of rhabdomyolysis and proteinuria, suppress production of the kidney protective antioxidant coenzyme Q10 and have other pleiotropic effects (10).These unintended adverse kidney effects was also indicated in previous studies. In a large scale, randomized controlled trial compared high dose (20 mg) rosuvastatin with placebo in almost 18000 patients. The results showed a non-significant increase of 19% in acute kidney injury (11).The non-significant risk increased further to 35% when the endpoint also comprised doubling of serum Cr. The non-significant risk increased further to 35% when the endpoint also comprised doubling of serum Cr (11). Moreover, a population based investigation of more than two million patients also detected that statin use was associated with a greater than 50% increase in risk of acute kidney injury, with evidence of raised risk within the first year of statin administration, and a dose-response impact (12).A retrospective analysis of statin use and hospital admission for acute kidney injury was conducted recently. The analysis is on large size, allows the detection of differences in a relatively uncommon outcome which many randomized controlled trials are underpowered to find. Interestingly, the authors selected a high versus low-potency statin rather than statin versus no statin administration, confirming that all participants had an indication for statin treatment (13).Mechanisms of renal toxicity by statins are ill-understood and potentially comprise of interruption of a wide variety of metabolic functions including membrane glycoprotein composition and fluidity, chloride channel activation and impaired mitochondrial function by reduced ubiquinone synthesis that might render lipoproteins more vulnerable to oxidation injury.Renal injury associated with the use of statins is frequently due to associated rhabdomyolysis producing acute tubular necrosis (14).

## 6. Conclusions

In the present study we found, atorvastatin with a doses of 150 mg/kg/day for 7 days was nephrotoxic for rats. While lower doses at 10 mg/kg/day or 50 mg/kg/day for 7 days was not accompanied by renal injury. These findings imply further attention to the administration of higher doses of atorvastatin in clinical conditions.

## Acknowledgements

This study was extracted from residential thesis of Zahra Hasanpour, which was conducted by a grant from Isfahan University of Medical Sciences.

## Authors’ contribution

ZH, HN and MN conducted the research. AA analyzed the data. HN prepared the primary draft. MRK edited the manuscript.

## Conflicts of interest

The authors declared no competing interests.

## Funding/Support

None to be declared.
